# The relationship of sex and sexual orientation to self-esteem, body shape satisfaction, and eating disorder symptomatology

**DOI:** 10.3389/fpsyg.2013.00887

**Published:** 2013-11-27

**Authors:** Chetra Yean, Erik M. Benau, Antonios Dakanalis, Julia M. Hormes, Julie Perone, C. Alix Timko

**Affiliations:** ^1^Department of Psychology, University of PennsylvaniaPhiladelphia, PA, USA; ^2^Department of Behavioral and Social Sciences, University of the SciencesPhiladelphia, PA, USA; ^3^Department of Brain and Behavioral Sciences, University of PaviaPavia, Italy; ^4^Department of Counseling and Psychological Services, West Chester UniversityWest Chester, PA, USA; ^5^Department of Psychology, Towson UniversityTowson, MD, USA

**Keywords:** sexual orientation, body image, body dissatisfaction, drive for muscularity, drive for thinness, eating disorder, self-esteem, gender differences

## Abstract

There is increasing interest in understanding what role, if any, sex and sexual orientation play in body dissatisfaction, its correlates to distress, and its relationship to disordered eating. The goals of the present study were to examine: (a) differences in sex and sexual orientation in internalization of societal pressure to modify physical appearance, components of body image dissatisfaction, self-esteem, and eating disorder symptomatology and (b) whether the internalization-eating disorder symptomatology was mediated by the different components of body image dissatisfaction and low self-esteem. The present data support several key trends in the literature: men generally reported less body dissatisfaction, internalization of socio-cultural standards of beauty, drive for thinness, and disordered eating, but a greater drive for muscularity than women; results also indicated that different components of body image dissatisfaction and low self-esteem partially mediated the relationship between internalization and eating disorder symptomatology. Gay men reported significantly more body dissatisfaction, internalization, eating disorder symptomatology, drive for thinness, and drive for muscularity than heterosexual men. Compared to heterosexual women, lesbians reported increased drive for muscularity, lower self-esteem, and lower internalization; however, they did not significantly differ on body dissatisfaction, drive for thinness or disordered eating. Correlation coefficients between body shape dissatisfaction and several aspects of mental distress were significantly larger for gay men than heterosexual men; the same coefficients did not differ between lesbian women and heterosexual women. Results of path analyses indicated that the relationship between internalization and disordered eating differs for gay and heterosexual men but not for lesbian and heterosexual women. These results call attention to lesbians as a generally understudied population.

## Introduction

There has been increasing interest in the risk of eating disorder symptomatology in lesbian, gay, and bisexual (LGB) populations. Despite many advances in the understanding of eating and body-related disorders in affluent Western countries, they continue to be an unresolved health problem, particularly for the LGB population (Morrison et al., 2004; Blashill, 2011). According to the socio-cultural model of body dissatisfaction (Stice, 1994), individuals who are regularly exposed to media messages with a strong emphasis on physical appearance are more likely to endorse these messages as personally relevant; that is, they are more likely to internalize the ideal body shape portrayed in the media. Endorsement of cultural body shape ideals and the values associated with them indicates that these ideals can become a reference point against which individuals judge their body; self-worth becomes contingent on meeting them (Fitzsimmons-Craft, 2011; Dakanalis and Riva, 2013). Among both sexes, internalization strongly predicts body dissatisfaction and low self-esteem. Both constructs have been found to be related to disordered eating (Stice and Shaw, 2002; Fitzsimmons-Craft, 2011; Dakanalis et al., 2014) and are recognized as two of the most important psychopathological characteristics of the development and maintenance of all forms of eating disturbances (Stice and Shaw, 2002; Cooper and Fairburn, 2011; Dakanalis et al., 2014). According to the socio-cultural model, men and women who internalize the cultural standards of beauty are more vulnerable to developing high levels of body dissatisfaction than those who do not, and, in turn, are more likely to engage in harmful behaviors in an attempt to control and modify their appearance according to what societal pressures dictate (Stice and Shaw, 2002).

While there is ample evidence supporting the socio-cultural model, direct measurement of its variables is increasingly complex when additional demographic factors (such as age, sex, ethnicity, nationality, socioeconomic status, and sexual orientation) are taken into account (Morrison et al., 2004; Soh et al., 2006; Grogan, 2008; Levine and Murnen, 2009; Blashill, 2011). Thus, the salience and impact of certain aspects of the model may vary for different populations. For example, measures of body image tend to center on a drive for thinness, which women typically endorse at higher rates than men; however, when assessing drive for muscularity, boys and men are more likely to report dissatisfaction with their body (Cohane and Pope, 2001; Grogan and Richards, 2002; Bergeron and Tylka, 2007).

Historically, it was hypothesized that gay men may be as affected as heterosexual women by socio-cultural pressures and therefore equally at risk for body dissatisfaction and consequent development of disordered eating (Grogan, 2008; Li et al., 2010; Blashill, 2011). In line with this hypothesis, gay men report more behavioral symptoms indicative of eating disorders than heterosexual men. In the United States, 14–42% of individuals with eating disorders are estimated to be gay and bisexual men (e.g., Carlat et al., 1997; Russell and Keel, 2002; Feldman and Meyer, 2007), while comprising only 0.5–4.0% of the U.S. population (Herzog, 2011). Several explanations have emerged as to why gay men are more likely to report eating and body image disturbances. Some of the most common arguments are framed within objectification theory (Fredrickson and Roberts, 1997), wherein gay men and heterosexual women are more likely to treat themselves as objects to be evaluated on the basis of physical appearance (i.e., self-objectification) which, in turn, increases their vulnerability to eating and body-related disturbances (e.g., Kozak et al., 2009; Wiseman and Moradi, 2010). Others have suggested that gay men have heightened intrasexual competition compared to heterosexual men or lesbian women (Morrison et al., 2004; Li et al., 2010), leading to an increased focus on appearance as a means to attract potential partners. It is also possible that gay men are more aware of and/or willing to disclose body or eating concerns than heterosexual men, thereby leading to a bias in the estimation of prevalence rates of disordered eating and body dissatisfaction in men (Dakanalis and Riva, 2013; Jankowski et al., 2013).

Despite the increasing interest in the nature of body dissatisfaction and the prevalence and mechanisms underlying weight and eating related disturbances in LGB individuals, research has primarily focused on gay men; comparatively little research has been devoted to lesbian women (Morrison et al., 2004). In the limited body of research comparing lesbian and heterosexual women, quantitative findings have been inconsistent. Some suggest that lesbian women may be “protected” from traditional, heteronormative pressures to be thin, and subsequently report less body dissatisfaction and disordered eating (Kozee and Tylka, 2006; Peplau et al., 2009). A recent meta-analysis, on the other hand, indicates that heterosexual and lesbian women do not significantly differ from each other in terms of body dissatisfaction (Morrison et al., 2004). A component of sociocultural models of disordered eating (Stice, 1994; Thompson et al., 2004) as well as objectification theory (Fredrickson and Roberts, 1997) is the pursuit of positive evaluation and attraction by others (i.e., to appear “attractive”). It is thought that the higher prevalence of disordered eating and body dissatisfaction among women is driven by the desire to appear attractive to men, however, research with lesbian women indicates pursuing a male partner is not necessary for body dissatisfaction to develop (Kozee and Tylka, 2006; Peplau et al., 2009). While lesbian culture is generally considered to be a feminist and body-positive social influence, and thus protective of disordered eating (e.g., by rejecting heteronormative behavior) (Kozee and Tylka, 2006), lesbians with diagnosed eating disorders report that issues related to sexuality (e.g., coming out) may negate the protectiveness of this positive social influence (Jones and Malson, 2013).

In addition to differences in prevalence of body dissatisfaction and eating disorder symptomatology based on sexual orientation, there are qualitative differences in the types of body ideals that gay and heterosexual men and women pursue. While gay men and heterosexual women both report heightened drive for thinness (Hunt et al., 2012), gay men additionally report increased drive for muscularity, a trait shared with heterosexual men (Yelland and Tiggemann, 2003; Duggan and McCreary, 2004; Brennan et al., 2012). Thus, regardless of sexual orientation, men report elevated preoccupation with enhancing musculature which is also associated with maladaptive weight/shape control behaviors (e.g., Yelland and Tiggemann, 2003; Dakanalis et al., 2013a). There is less research regarding body ideals of lesbian women, but studies suggest that body ideals tend not to vary from heterosexual women (Feldman and Meyer, 2007; Peplau et al., 2009; Koff et al., 2010). In terms of appearance, many lesbians may desire to appear “butch” (which is similar to, but not the same as, a “masculine” appearance; Case, 1999), which is largely related to social identity and wanting to “feel authentic” (Cogan, 1999; Levitt and Hiestand, 2004). It is not established whether traditional measures of appearance or body dissatisfaction adequately assess the social-identity aspect for lesbians or the type of appearance lesbians would prefer.

There were two main goals of the present study. The first goal was to extend the extant literature and to comprehensively examine male and female body and appearance ideals and their relation with socio-cultural pressure, global self-esteem, and disordered eating. We used measures that address different components of body image dissatisfaction (i.e., overall body dissatisfaction, desire to be thin, desire to be muscular) in heterosexual and gay men and women. We hypothesized that, compared to heterosexual men, gay men would report higher rates of body dissatisfaction, increased drive for muscularity and thinness, and increased eating disorder symptomatology. We also hypothesized that, compared to heterosexual women, lesbian women would report higher drive for muscularity and lower internalization scores but would not significantly differ on other measures.

The second goal of the current study was to examine a more comprehensive model than has been previously explored, which includes both links of internalization of sociocultural standards of beauty to eating disorder symptomatology, and the mediating roles of the different components of body image dissatisfaction and low self-esteem. We expected that internalization would lead to different components of body image dissatisfaction (i.e., overall body dissatisfaction, desire to be thin, desire to be muscular) and lower self-esteem, which both in turn contribute to disordered eating. However, given the paucity of research investigating sex and particular sexual orientation differences in the strength of these relationships between variables (Fitzsimmons-Craft, 2011), it is unclear how well these relationships fit men and women of different sexual orientations, and if the relationship between internalization and disordered eating is fully or partially mediated by the different components of body image dissatisfaction and low self-esteem (i.e., presence of a direct relation from internalization to disordered eating). Although exploring mediation was the primary goal, we not have specific predictions regarding the overall fit, specific model paths, or the type of mediation.

## Method

### Participants and procedure

Participants were recruited from three urban universities and their surrounding areas in the mid-Atlantic United States. Community participants were recruited from organizations serving specific ethnic groups and sexuality groups and via the Internet to assure inclusion of traditionally underrepresented minorities in the sample of respondents. All participants completed the study's measures online. The use of online surveys generally does not change the quality of results compared to paper-and-pencil (Lewis et al., 2009).

Nine hundred and fifty (950) participants initiated the survey and 702 completed it (74% response rate). For one of the universities, 329 participants were compensated with course credit for their completion of the questionnaire. We excluded respondents under 18 years of age (*n* = 6) and those who identified as transgender (*n* = 3). Therefore, the final sample consisted of 693 respondents and included 246 men (35.5%) and 447 women (64.5%). The appropriate ethical review board at each institution approved the study. The final sample included 187 respondents from the community and 506 undergraduate students from the three campuses combined into a single subsample^1^.

Sexual orientation was measured using a seven-point Likert-type scale based on the Kinsey et al. (1948) scale (described further below). We collapsed sexual orientation into a trinary: heterosexual (0 and 1 on the scale), bisexual (2, 3, and 4 on the scale), and exclusively gay (5 and 6 on the scale). This classified 130 men (53%) as heterosexual, 15 as bisexual (6%), and 101 as gay (41%); there were also 361 heterosexual women (80%), 48 bisexual women (11%), and 38 gay women (9%). Thus, 47% of men (*n* = 116) and 19% of women (*n* = 86) were classified as gay or bisexual. Despite our attempts at even distribution of sex and sexual orientation, heterosexual women were significantly overrepresented in this sample (χ^2^ = 59.87, *p* < 0.001, Φ = 0.29). The sample was largely Caucasian (*n* = 492, 71.0%) and the remainder was ethnically diverse: 7.4% were African American (*n* = 51), 4.6% were Hispanic (*n* = 32), 13.4% Asian/Pacific Islander (*n* = 93), 3.3% Native American (*n* = 23), 0.3% responded with “Other” or did not respond to this question (*n* = 2). Respondents were generally younger adults with a mean age of 21.23 years (*SD* = 5.56, *Mdn* = 21.0, range = 18–60); 94.2% of the sample was ≤30 years old. The community sample was significantly older (*M* = 25.02, *SD* = 8.46, *Mdn* = 22.0, range = 18–60) than the university sample (*M* = 19.83, *SD* = 2.95, *Mdn* = 19.0, range = 18–52) (*U* = 20307.00, *Z* = −11.75, *p* < 0.001, *r* = 0.45). *U*-tests indicate that the distribution of Body Mass Index (*BMI* = kg/m^2^) categories did not statistically differ between university and community (*p* = 0.079) and men and women (*p* = 0.233). A Kruskal–Wallis test indicated no main effect of sexual orientation on BMI categories for men (*p* = 0.215), but a significant main effect for women [χ^2^_(2, *n* = 447)_ = 6.42, *p* = 0.003]. Using *post-hoc* Mann–Whitney *U*-tests using Holm's sequential correction (described further below), lesbians had significantly more respondents in the overweight and obese categories (47%) than heterosexual women (19%) (*U* = 5150.50, *Z* = −3.097, *p* = 0.002, *r* = 0.155); bisexual women did not significantly differ from heterosexual (*p* = 0.049) or lesbian women (*p* = 0.561).

### Measures

#### Demographics

The *Kinsey Heterosexual-Homosexual Likert-type Scale* (Kinsey et al., 1948) was used to assess sexual orientation. Participants indicated their self-identified sexual orientation on a scale of zero to six: each item on the scale was labeled according to the original version, where zero indicated “exclusively heterosexual,” three indicated “equally heterosexual and homosexual” and at six indicated “exclusively homosexual.” While there are many ways to measure sexual orientation (Sell, 1997), the Kinsey scale remains a valid and parsimonious instrument, particularly for online surveys in Western cultures (Drucker, 2012). As noted, we created three categories for sexual orientation: heterosexual (0 and 1 on the scale), bisexual (2, 3, and 4 on the scale), and exclusively gay (5 and 6 on the scale). Participants also completed: the 36-item *Body Shape Questionnaire* (BSQ; Cooper et al., 1987) which assesses overall satisfaction with one's body shape; the 26-item *Eating Attitudes Test-26* (EAT; Garner and Garfinkel, 1979) which quantifies eating disorder symptomatology; the 7-item *Drive for Thinness Scale* from the Eating Disorder Inventory-II (DFT; Garner, 1991) which specifically assesses an individual's desire to be thin; the 15-item *Drive for Muscularity Scale* (DFM; McCreary and Sasse, 2000) which captures an individual's desire for muscularity; the 10-item *Rosenberg Self-Esteem Scale* (RSES; Rosenberg, 1965) which assesses global self-esteem; and the 9-item general internalization subscale of the *Sociocultural Attitudes Towards Appearance Scale-3* (SATAQ) (Thompson et al., 2004) which assesses how media and social influences impact an individual's perception and opinion of appearance. All scales had excellent internal consistency for the whole sample (all Cronbach's αs > 0.90), and acceptable consistency for subsamples of gender and sexual orientation (all αs > 0.78). Participants also provided height and weight to calculate BMI. Means and standard deviations for each included measure are found in Table 1.

**Table 1:** **Descriptive statistics for each variable of interest**.

	**Entire sample (V = 693)**	**Men (All) (*n* = 246)**	**Heterosexual men (*n* = 130)**	**Gay/Bisexual men (*n* = 116)**	**Women (All) (*n* = 447)**	**Heterosexual women (*n* = 361)**	**Gay/Bisexual women (*n* = 86)**
BSQ	84.53 (36.42)	69.18 (28.98)	63.43 (23.79)	75.63 (32.78)	92.97 (37.35)	92.63 (35.97)	94.41 (42.85)
DFT	4.53 (5.93)	2.61 (4.45)	1.55 (2.65)	3.79 (5.61)	5.59 (6.36)	5.48 (6.28)	6.06 (6.70)
DFM	32.16 (13.31)	41.41 (13.68)	42.83 (13.28)	39.83 (14.00)	27.06 (9.95)	26.24 (9.16)	30.55 (12.17)
SATAQ	27.16 (8.78)	26.05 (8.46)	24.95 (7.96)	27.29 (8.85)	27.77 (8.91)	28.33 (8.66)	25.40 (9.57)
EAT	9.14 (10.93)	6.95 (8.97)	5.06 (5.62)	9.06 (11.30)	10.35 (11.71)	9.90 (11.15)	12.24 (13.72)
RSES	20.86 (6.00)	20.89 (5.85)	21.08 (5.84)	20.67 (5.89)	20.84 (6.08)	21.37 (5.80)	18.65 (6.78)
BMI	23.48 (5.06)	23.72 (4.28)	24.12 (3.91)	23.28 (4.62)	23.34 (5.43)	22.87 (4.84)	25.35 (7.12)

Mean (SD); BSQ, Body Shape Questionnaire; DFT, Drive for Thinness Scale from the Eating Disorder Inventory-II; DFM, Drive for Muscularity; SATAQ, Sociocultural Attitudes Toward Appearance Questionnaire; EAT, Eating Attitudes Test; RSES, Rosenberg Self-Esteem Questionnaire; BMI, Body Mass Index (kg/m^2^); 5 men (4 heterosexual) and 10 women (8 heterosexual) did not report their height and/or weight to calculate BMI.

#### Statistical analysis

We used the total and subscale scores of the measures described above as the dependent variables in all analyses. Due to an administration error, item no. 27 on the SATAQ (“I do not try to look like the people on TV”) was lost for all participants. Because all other items in the SATAQ's internalization subscale were administered properly, we replaced the lost data using mean imputation from the remaining responses on the scale. There was a one-tailed, non-normal distribution across each variable and in each subgroup as the Kolmogorov-Smirnov and Shapiro-Wilk tests were significant for each questionnaire (*p*s < 0.001); thus the assumption of normality for analysis of variance (ANOVA) was violated. To assess differences between the heterosexual, gay, and bisexual samples, we conducted a series of Mann–Whitney *U*-tests (a nonparametric equivalent to *t*-tests). First, we collapsed sexual orientation into a binary (gay/bisexual and heterosexual). We further compared sexual orientation as a trinary: bisexuals were compared to exclusively gay and exclusively heterosexual individuals; exclusively gay individuals were also compared to heterosexuals. To control for Type 2 error, we utilized Holm's (1979) sequential correction; this method is preferable to a traditional Bonferroni correction as it does not change the *p*-value, but instead provides critical values for significance (Aickin, 1996). In order to be considered significant, the smallest *p*-value must be less than 0.008 (0.05/6), the second smallest less than 0.01 (0.05/5), the third less than 0.0125 (0.05/4), the fourth less than 0.0167 (0.05/3), the fifth less than 0.025 (0.05/2), and finally the sixth less than 0.05 (0.05/1). We conducted Spearman Rank-Order correlations (*r*_*s*_) between each variable stratified by sex and binary sexual orientation. Sexual orientation was collapsed into a binary to increase power and because bisexual respondents generally did not differ from exclusively gay members of the same sex. We conducted Fisher r-to-z transformations to assess if the correlation coefficients (*r*_*s*_) were significantly larger between sexual orientations of the same sex.

We conducted a path analysis using Mplus version 6.1 (Muthén and Muthén, 2010) to determine whether there was a good fit to the data for the hypothesized model linking internalization to disordered eating behaviors through different components of body image dissatisfaction and low self-esteem. The procedure was conducted first for men and then for women. Total scores on the measures served as the observed variables in the model. Because pre-analysis of the data revealed evidence for non-normality (see above), we used robust maximum likelihood estimation (Byrne, 2011). We determined the adequacy of model fit by four indices recommended by Byrne (2011): the Comparative Fit Index (CFI), the Tucker-Lewis Index (TLI), the Standardized Root-Mean Square Residual (SRMR), and the Root Mean Square Error of Approximation (RMSEA). CFI and TLI values ≥ 0.95, SRMR values ≤ 0.08 and RMSEA values ≤ 0.06 indicate a good representation of the data (Byrne, 2011). We also specified Mplus to identify modification indices (MI) above 5.0, as there may have been significant paths between variables that were not hypothesized (e.g., from different components of body image dissatisfaction to low-self-esteem) and examined in the model (Muthén and Muthén, 2010). In order to obtain the most parsimonious and accurate representation of the data, we planned to trim paths that were not significant and add paths not originally specified (MIs > 5.0) but impacted the fit of the model to the data, as recommended by Byrne (2011). The Satorra-Bentler scaled chi-square difference test (S-BΔχ^2^) was used to compare these nested models (Byrne, 2011). As testing mediation using bootstrap procedure has been recommended (Mackinnon, 2011), the (final) structural model was run with 1000 bootstrap samples to examine the significance of indirect effects. The bootstrap standardized indirect path coefficients and 95% bias-corrected confidence intervals (95% CI), were reported. Indirect effects are significant if their 95% CI does not include zero (Mackinnon, 2011). In order to determine whether the structural paths of the (final) model were similar or different across sexual orientations groups, two multiple-group analyses were performed. Using this method, if the invariant model, in which the structural paths' values were constrained to be equal first for heterosexual and gay/bisexual men and then for heterosexual and gay/bisexual women (given the minimum 10:1 participants-to-parameter ratio needed to examine a model, sexual orientation was collapsed into a binary; Muthén and Muthén, 2010) did not differ in fit from the free model (i.e., without constrictions), then the structural path coefficients would not be significantly different across sexual orientation groups. The S-BΔχ^2^ was used to compare model fit (Byrne, 2011).

## Results

### Sex differences

The results of Mann–Whitney *U*-tests comparing men and women on the study's variables can be seen in Table 2. Men endorsed significantly more drive for muscularity, which was the largest effect size in any comparison within the sample (*r* = 0.52). Women endorsed significantly more body shape dissatisfaction, drive for thinness, internalization, and disordered eating symptomatology as measured by the EAT. Small-to-medium effect sizes were found in the comparisons of body shape dissatisfaction (*r* = 0.32) and drive for thinness (*r* = 0.25), a small effect size was found for eating disorder symptomatology (*r* = 0.17), and a negligible effect size was found for the internalization subscale (*r* = 0.09). Men and women did not significantly differ on a measure of self-esteem. Each of the above significances was maintained using Holm's correction.

**Table 2:** **Results of Mann–Whitney *U*-tests comparing men (*n* = 246) and women (*n* = 447)**.

	**Men median**	**Women median**	***Z* (*U*)**	**Sig**.	**Effect size (*r*)**
BSQ	62.00	89.00	−8.42 (33,748)	<0.001	0.320
DFT	0.00	3.00	−6.65 (38,808)	<0.001	0.253
DFM	41.00	25.00	−13.64 (20,595)	<0.001	0.518
SATAQ	27.00	28.13	−2.45 (48,800)	0.014	0.093
EAT	4.00	6.00	−4.35 (44,042)	<0.001	0.165
RSES	20.50	21.00	−0.02 (54,943)	0.988	0.001

BSQ, Body Shape Questionnaire; DFT, Drive for Thinness Scale from the Eating Disorder Inventory-II; DFM, Drive for Muscularity Scale; SATAQ, Sociocultural Attitudes Toward Appearance Questionnaire; EAT, Eating Attitudes Test; RSES, Rosenberg Self-Esteem Scale; U-values and medians are rounded.

### Sexual orientation

As shown in Table 3, compared to heterosexual men, exclusively gay men reported significantly more body shape dissatisfaction and eating disorder symptomatology; these significances were maintained after Holm's correction. Exclusively gay men reported lower and drive for thinness and internalization; these differences were marginally significant. Gay and heterosexual men did not significantly differ on self-esteem or drive for muscularity. The largest effect size in these comparisons was for body shape dissatisfaction (*r* = 0.216). Bisexual men reported less body shape dissatisfaction (*mdn* = 49.0, *M* = 63.20, *SD* = 29.03) than exclusively gay men (*mdn* = 69.0, *M* = 77.48, *SD* = 33.04), which was a trend level association (*U* = 529.00, *Z* = −1.880, *p* = 0.060, *r* = 0.123). There were no other significant differences between bisexual men and exclusively gay or exclusively heterosexual men (all *p*s > 0.1).

**Table 3:** **Results of Mann–Whitney *U*-tests comparing the variables of interest between gay men (*n* = 116) and heterosexual men (*n* = 130)**.

	**Gay/Bisexual men median**	**Heterosexual men median**	***Z* (*U*)**	**Sig**.	**Effect size (*r*)**
BSQ	67.00	58.00	−2.83 (5962)	0.005^*^	0.181
DFT	0.00	0.00	−2.17 (6436)	0.030	0.138
DFM	38.00	42.00	−1.83 (6518)	0.066	0.117
SATAQ	3.13	2.88	−2.87 (5945)	0.031	0.183
EAT	5.00	3.00	−2.15 (6342)	0.004^*^	0.137
RSES	20.00	21.00	−0.43 (72,978)	0.663	0.028

* Maintains significance using Holm's sequential correction. BSQ, Body Shape Questionnaire; DFT, Drive for Thinness Scale from the Eating Disorder Inventory-II; DFM, Drive for Muscularity Scale; SATAQ, Sociocultural Attitudes Toward Appearance Questionnaire; EAT, Eating Attitudes Test; RSES, Rosenberg Self-Esteem Scale; U-values and medians are rounded.

As shown in Table 4, lesbian and bisexual women reported significantly more drive for muscularity and significantly lower internalization of the thin ideal and self-esteem. Both of these differences were maintained using Holm's correction. There were small effect sizes for both of the above significant differences. Lesbian and bisexual and heterosexual women did not significantly differ on body shape dissatisfaction, drive for thinness, or eating disorder symptomatology. When exclusively gay women were compared with exclusively heterosexual women, the comparison of self-esteem was no longer significant (*p* = 0.188); there were no other changes in significance and all effect sizes remained small (*r*s > 0.1 and < 0.2).

**Table 4:** **Results of Mann–Whitney *U*-tests comparing the variables of interest between lesbian women (*n* = 86) and heterosexual women (*n* = 361)**.

	**Lesbian/Bisexual women median**	**Heterosexual women median**	***Z* (*U*)**	**Sig**.	**Effect size (*r*)**
BSQ	79.00	90.00	−0.00 (15,521)	0.998	0.000
DFT	3.00	3.00	−0.51 (14,980)	0.607	0.024
DFM	28.50	24.00	−2.89 (12,416)	0.004^*^	0.137
SATAQ	25.87	29.25	−2.61 (12,716)	0.009^*^	0.123
EAT	6.00	6.00	−1.13 (14,305)	0.257	0.054
RSES	19.00	22.00	−3.47 (11,797)	0.001^*^	0.164

* Maintains significance using Holm's sequential correction; BSQ, Body Shape Questionnaire; DFT, Drive for Thinness Scale from the Eating Disorder Inventory-II; DFM, Drive for Muscularity Scale; SATAQ, Sociocultural Attitudes Toward Appearance Questionnaire; EAT, Eating Attitudes Test; RSES, Rosenberg Self-Esteem Scale; U-values and medians are rounded.

Bisexual women reported less drive for muscularity (*mdn* = 25.5, *M* = 28.04, *SD* = 10.89) than exclusively gay women (*mdn* = 31.0, *M* = 33.71, *SD* = 13.09, *U* = 672.50, *Z* = −2.084, *p* = 0.037, *r* = 0.225); however, this association is not considered significant using Holm's correction. Bisexual women reported significantly lower self-esteem (*mdn* = 17.5, *M* = 17.75, *SD* = 6.62) than exclusively heterosexual women (*mdn* = 22.0, *M* = 21.37, *SD* = 5.80, *U* = 5827.5, *Z* = −3.691, *p* < 0.001, *r* = 0.183), which is significant using Holm's correction. Thus, bisexual women reported the lowest self-esteem of the three sexual orientation groups, while exclusively heterosexual and gay women did not significantly differ. There were no other significant or trend-level differences between bisexual, and exclusively heterosexual or exclusively gay women (all *p*s > 0.1).

### Correlations between variables

Table 5 presents correlations among all variables for separated by sex and sexual orientation. Also noted in this table are any significant differences in strength of correlation coefficients. The only significant differences between gay and heterosexual individuals' correlation coefficients were observed in the male sample: body shape dissatisfaction was more strongly correlated to eating disorder symptomatology, drive for thinness, and self-esteem in gay men, than heterosexual men. In addition, drive for thinness correlated with eating disorder symptomatology more strongly for gay men than heterosexual men.

**Table 5:** **Spearman Rank-Order Correlations between each variable stratified by sex and sexual orientation**.

	**BSQ**	**DFT**	**DFM**	**SATAQ**	**EAT**	**RSES**
**HETEROSEXUAL MEN (BELOW DASHES; *n* = 130) AND WOMEN (ABOVE DASHES; *n* = 361)**						
BSQ	−	0.801^***^	0.269^***^	0.565^***^	0.687^***^	−0.473^***^
DFT	0.613^***^	−	0.198^***^	0.477^***^	0.809^***^	−0.375^***^
DFM	0.271^**^	0.288^**^	−	0.230^***^	0.232^***^	−0.134^*^
SATAQ	0.376^***^	0.429^***^	0.463^***^	−	0.457^***^	−0.330^***^
EAT	0.462^***^	0.549^***^	0.404^***^	0.513^***^	−	−0.274^***^
RSES	−0.287^**^	−0.317^**^	−0.244^**^	−0.313^**^	−0.252^**^	−
**GAY/BISEXUAL MEN (BELOW DASHES; *n* = 116) AND WOMEN (ABOVE DASHES; *n* = 86)**						
BSQ	−	0.757^***^	0.231^*^	0.600^**^	0.671^***^	−0.504^***^
DFT	0.762^***^^a^	−	0.164	0.489^***^	0.845^***^	−0.409^***^
DFM	0.270^**^	0.152	−	0.185	0.262^*^	−0.12
SATAQ	0.509^***^	0.415^***^	0.310^**^	−	0.447^***^	−0.359^***^
EAT	0.695^***^^b^	0.742^***^^b^	0.263^**^	0.319^***^	−	−0.359^**^
RSES	−0.493^***^^a^	−0.442^***^	−0.115	−0.119	−0.366^***^	−

* p < 0.05;

** p < 0.01;

*** p < 0.001; Fisher r-to-z transformation reveals a significant difference between correlation coefficients of heterosexual and gay members of the same sex where

a p < 0.05,

b p < 0.01; BSQ, Body Shape Questionnaire; DFT, Drive for Thinness Scale from the Eating Disorder Inventory-II; DFM, Drive for Muscularity; SATAQ, Sociocultural Attitudes Toward Appearance Questionnaire; EAT, Eating Attitudes Test; RSES, Rosenberg Self-Esteem Scale.

### Path and multi-group analyses and test of mediation

**Figure 1:**
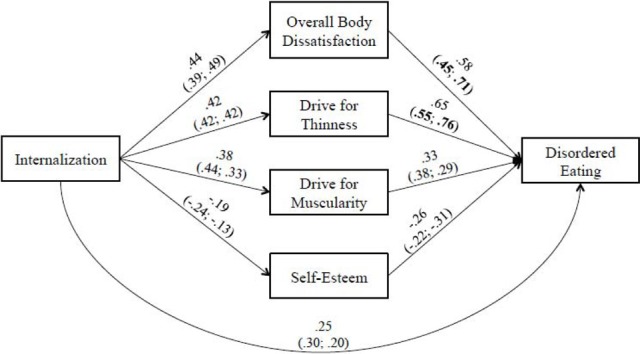
**Model of the relationship between internalization of socio-cultural standards of beauty and disordered eating for men**. Revised model with significant paths added. Path coefficients for the full sample of men as well as for the sub-samples of heterosexual (right side) and gay/bisexual men (left side) are presented (all *ps* < 0.05). The bold values within parentheses indicate a significant difference between heterosexual (right side) and gay/bisexual (left side) men.

An initial test of the hypothesized model in men resulted in a good fit to the data: *CFI* = 0.95, *TLI* = 0.96, *SRMR* = 0.07, *RMSEA* = 0.04, and all paths were significant (*ps* < 0.05). However, upon inspection of the MIs, we noted an unexpected path with a large MI (>5.0) in the model: the path from internalization to disordered eating, indicating that there is a direct relationship between these two variables This path was subsequently added and the model was re-evaluated. The revised model provided a significantly better fit than the original model, *CFI* = 0.96, *TLI* = 0.96, *SRMR* = 0.06, *RMSEA* = 0.04 without the added path [S-BΔχ^2^_(1, *N* = 246)_ = 13.6, *p* < 0.001], and, consequently, was retained. As can been seen in Figure 1, the internalization-disordered eating path is partially mediated (i.e., presence of a significant path from internalization to disordered eating; Mackinnon, 2011) by self-esteem and components of body image dissatisfaction (i.e., overall body dissatisfaction, desire to be thin, desire to be muscular). This was confirmed by the results of bootstrapping procedure (see Table 6), indicating that all indirect effects displayed in Figure 1 are significant. In other words, internalization led directly and indirectly (via the different components of body image dissatisfaction and low self-esteem) to disordered eating. In order to determine whether the structural paths illustrated in Figure 1 were similar or different for heterosexual and gay/bisexual men, multi-group analyses was performed. The difference in fit between the unconstrained and constrained model was significant [S-BΔχ^2^_(9, *N* = 246)_ = 38.7, *p* < 0.05], suggesting that one or more paths among the variables would be different across groups. Follow-up analyses indicated that the path coefficients from overall body dissatisfaction [S-BΔχ^2^_(1, *N* = 246)_ = 6.38, *p* < 0.01] and drive for thinness to [S-BΔχ^2^_(1, *N* = 246)_ = 5.94, *p* < 0.01] to disordered eating were responsible for the non-invariance. Both associations were stronger for gay/bisexual men than for heterosexual men (see Figure 1). Among heterosexual men, internalization predicted 19, 21, 22, and 12% of the variance in overall body dissatisfaction, drive for thinness, drive for muscularity and low self-esteem. Internalization, overall body dissatisfaction, drive for thinness, drive for muscularity and low self-esteem predicted 13, 23, 28, 20, and 11% of the variance in disordered eating. Among gay/bisexual men, internalization predicted 25, 21, 18, and 10% of the variance in overall body dissatisfaction, drive for thinness, drive for muscularity and low self-esteem. Internalization, overall body dissatisfaction, drive for thinness, drive for muscularity and low self-esteem predicted 11, 35, 39, 12, and 14% of the variance in disordered eating.

**Table 6:** **Mediation: examination of indirect effects and bias-corrected 95% confidence intervals (CIs)**.

	**Indirect effect (β)**	**95% CIs**
**MEN (*N* = 246) INDIRECT PATH**		
Internalization → Body dissatisfaction → Disordered eating	0.24^*^	0.161–0.282
Internalization → Drive for thinness → Disordered eating	0.27^*^	0.181–0.300
Internalization → Drive for muscularity → Disordered eating	0.12^*^	0.048–0.168
Internalization → Self-esteem → Disordered eating	0.05^*^	0.020–0.117
**WOMEN (***N*** = **447**) INDIRECT PATH**		
Internalization → Body dissatisfaction → Disordered eating	0.35^*^	0.235–0.382
Internalization → Drive for thinness → Disordered eating	0.36^*^	0.279–0.398
Internalization → Drive for muscularity → Disordered eating	0.04^*^	0.022–0.111
Internalization → Self-esteem → Disordered eating	0.10^*^	0.044–0.190

* p < 0.05.

**Figure 2:**
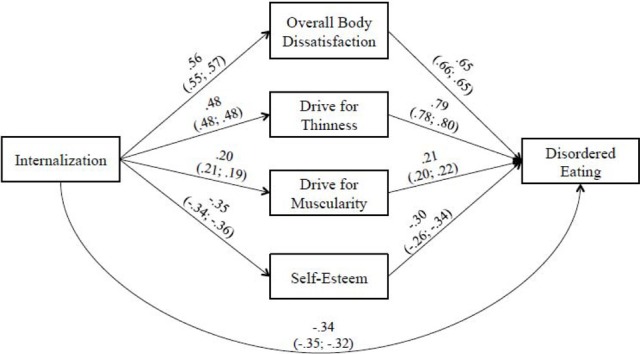
**Model of the relationship between internalization of socio-cultural standards of beauty and disordered eating for women**. Revised model with significant paths added. Path coefficients for the full sample of women as well as for the sub-samples of heterosexual (right side) and gay/bisexual women (left side) are presented (all *p*s < 0.01).

When the components of the hypothesized model were specified in the sample of women, the model provided a very good fit to the data (*CFI* = 0.98, *TLI* = 0.98, *SRMR* = 0.05, *RMSEA* = 0.05) and all paths were significant (*p*s < 0.01). When inspecting the MIs, we noted that the path from internalization to disordered eating had a value >5.0. When this path was added to the model, the results indicated that the revised model provided a significantly better fit than the original model [*CFI* = 0.98, *TLI* = 0.99, *SRMR* = 0.04, *RMSEA* = 0.03, S-BΔχ^2^_(1, *N* = 447)_ = 16.4, *p* < 0.001], and consequently the revised model was retained. As can been seen in Figure 2, the internalization-disordered eating is partially mediated by components of body image dissatisfaction (i.e., overall body dissatisfaction, desire to be thin, desire to be muscular) and self-esteem; all indirect effects displayed are significant (see Table 6). Furthermore, the difference in fit between the unconstrained and constrained model, was not significant, [S-BΔχ^2^_(9, *N* = 447)_ = 18.3, *p* > 0.05]; thus the structural path coefficients are similar across sexual orientation groups (i.e., heterosexual vs. gay/bisexual women). Among heterosexual women, internalization predicted 28, 24, 10, and 17% of the variance in overall body dissatisfaction, drive for thinness, drive for muscularity and low self-esteem. Internalization, overall body dissatisfaction, drive for thinness, drive for muscularity and low self-esteem predicted 17, 33, 39, 10, and 13% of the variance in disordered eating. Among gay/bisexual women, internalization predicted 27, 24, 9, and 16% of the variance in overall body dissatisfaction, drive for thinness, drive for muscularity and low self-esteem. Internalization, overall body dissatisfaction, drive for thinness, drive for muscularity and low self-esteem predicted 15, 33, 40, 11, and 16% of the variance in disordered eating.

## Discussion

The purpose of this study was to extend and synthesize extant research examining differences in sexual orientation in internalization of societal pressure to modify physical appearance, body image dissatisfaction, drive for muscularity, drive for thinness, internalization of societal pressure to modify physical appearance, self-esteem, and eating disorder symptomatology, and in their associations to predict eating disturbances in both men and women. Based on the postulation of the socio-cultural model and recent empirical findings (Stice, 1994; Stice and Shaw, 2002; Stice et al., 2007; Dakanalis et al., 2012, 2013a, 2014; Dakanalis and Riva, 2013), we also examined overall body dissatisfaction, desire to be thin, desire to be muscular, and low self-esteem as mediators of the internalization of sociocultural standards of beauty-disordered eating relationship. To accomplish these goals, we recruited a sample from three separate universities and the broader LGB community via online survey. Women in the sample reported significantly more body shape dissatisfaction, drive for thinness, internalization, and eating disorder symptomatology. Men reported significantly more drive for muscularity, a difference that had the largest effect size of any analysis in the sample. Men and women did not differ on a measure of self-esteem.

Compared to men, women reported more internalization of societal pressure to modify appearance, dissatisfaction with their body shape, and eating disorder symptomatology. The present results are thus in line with previous research that identifies women as the group most vulnerable to body image dissatisfaction and subsequent disordered eating (Striegel-Moore et al., 2009; Koff et al., 2010; Dakanalis et al., 2013a,b). These findings are not surprising as body shape dissatisfaction is recognized as a substantial risk factor for women's clinical and sub-clinical eating disorders (Stice and Shaw, 2002). Compared to women, men reported significantly more drive for muscularity, which is also consistent with previous research (Grogan and Richards, 2002; Duggan and McCreary, 2004; Bergeron and Tylka, 2007; Dakanalis and Riva, 2013; Dakanalis et al., 2013a). The present data further demonstrate that, compared to men, women report more dissatisfaction with their bodies, drive for thinness, and eating disorder symptomatology. Men, on the other hand, desire increased muscle mass. The results indicate that different components of body image dissatisfaction and low self-esteem partially mediated the relationship between internalization and eating disorder symptomatology, suggesting the same underlying mechanisms translated the socio-cultural pressure to eating disorder symptomatology in both sexes. If future research confirms these findings, then eating disorder prevention programs should add a focus to self-esteem.

Consistent with previous research, and supporting our hypotheses, gay men reported significantly more body shape dissatisfaction, internalized pressure to modify physical appearance, and eating disorder symptomatology (Yelland and Tiggemann, 2003; Duggan and McCreary, 2004; Olivardia et al., 2004; Blashill, 2011; Jankowski et al., 2013). However, counter to our hypotheses and previous research that suggests gay men, rather than heterosexual men, desire a paradoxically thin, yet muscular body (Yelland and Tiggemann, 2003; Duggan and McCreary, 2004; Olivardia et al., 2004; Blashill, 2011; Hunt et al., 2012), gay men reported a trend-level *decrease* in drive for muscularity compared to heterosexual men, and an increase in drive for thinness that was marginally significant, or not statistically significant using Holm's correction. Thus, both heterosexual- and gay-identified men reported similar amounts of desire for thinness and muscularity. It may be that the age composition and/or social environment of the present sample reduced the effect: that is, the desire for increased muscle mass along with a thinner body reduces with age and/or this effect may be larger in male undergraduates. Non-student and/or older respondents may have reduced the overall scores on this response. Additionally, while drive for thinness and drive for muscularity were significantly correlated for heterosexual but not gay men, the correlation coefficients did not significantly differ between the two groups. Therefore, the present data cannot fully support previous findings that the drive for a paradoxically thin, yet muscular, body is limited to gay men and not heterosexual men (e.g., Yelland and Tiggemann, 2003). It should be noted that while these desires did not substantially differ between the two groups, the difference in a questionnaire evaluating explicit body shape dissatisfaction was much higher for gay men. This supports the idea that gay men are more likely to recognize or identify body shape dissatisfaction, and/or disclose these feelings (Dakanalis and Riva, 2013; Jankowski et al., 2013).

The correlations between body dissatisfaction and drive for thinness, eating disorder symptomatology, and self-esteem were significantly larger for gay and bisexual men, as was the correlation coefficient between eating disorder symptomatology and drive for thinness. Likewise, the paths from body dissatisfaction and drive for thinness to disordered eating were significantly different between gay/bisexual men and heterosexual men. A larger percentage of the variance in disordered eating was explained by both of these variables in gay/bisexual men. In conjunction with the results of the *U*-tests, this indicates that gay and bisexual men are not only more dissatisfied with their body than heterosexual men, but that this dissatisfaction is also more strongly associated with a variety of distress and disordered eating. As body-shape dissatisfaction increased for the gay men in the present sample, they were more likely than heterosexual men to desire a thin body than a muscular one, or vice-versa. It is unlikely that these associations are due to differences in self-esteem, as heterosexual and gay men did not differ on a measure of global self-esteem. These results suggest that men, regardless of sexual orientation, may be susceptible to pressures to simultaneously increase muscle mass and reduce body fat, possibly in an attempt to make their muscle mass more visible (Dakanalis and Riva, 2013). However, drive for thinness has a larger impact on disordered eating for gay and bisexual men: they are more likely to engage in disordered eating to achieve the weight loss than heterosexual men.

Similar to previous samples of gay and bisexual men (Hunt et al., 2012), drive for thinness negatively correlated with self-esteem; however, in contrast to previous studies (Yelland and Tiggemann, 2003; Hunt et al., 2012), drive for muscularity did not. Consistent with prior samples of heterosexual men (Olivardia et al., 2004), there were significant negative correlations between self-esteem and both drive for thinness and drive for muscularity. These findings suggest that, for gay and bisexual men, drive for thinness may be a more powerful determinant of self-esteem, body image dissatisfaction, and disordered eating than drive for muscularity; whereas for heterosexual men, both are equally powerful determinants of self-esteem. Likewise, the results of the path analysis indicating that body dissatisfaction and drive for thinness account for more variance in disordered eating highlight the important role of drive for thinness in gay men. Further research comparing heterosexual and gay men is warranted to clarify the above findings.

As hypothesized, lesbian women in the present sample reported significantly more drive for muscularity and lower internalization of socio-cultural standards of beauty than heterosexual or bisexual women. The differences in drive for muscularity were the larger of the effect sizes in the comparisons of lesbian women to both bisexual and heterosexual women, respectively. To our knowledge, no previous research has directly compared drive for muscularity in heterosexual, bisexual and lesbian women. While lesbians generally report similar levels of body dissatisfaction to heterosexual women (Morrison et al., 2004), they may feel more of a drive to look “butch” to appear “authentically lesbian” (e.g., Levitt and Hiestand, 2004). It has been previously suggested that many lesbians pursue a more masculine, or “butch,” aesthetic to exhibit a renouncement of heteronormative feminine ideals and/or are comfortable adopting some traditionally masculine traits (Beren et al., 1997; Case, 1999). Consequently, the increased drive for muscularity in this sample may not be a facet of body dissatisfaction as it is traditionally considered (i.e., to appear attractive) (Stice and Shaw, 2002; Morrison et al., 2004), but rather is an index of a desired social identity. The present data only partially support this idea: the internalization scores were significantly lower, and drive for muscularity was significantly higher for the sample of gay and bisexual women than for heterosexual women, but the two measures significantly correlated only for heterosexual women. However, no correlation coefficient significantly differed between the two groups of women, suggesting that the correlations of these variables between the two populations are similar. The results of the path analysis, in which there was no significant difference between the invariant and free model, support the hypothesis that body dissatisfaction, drive for thinness, and drive for muscularity account for comparable amounts of variance in the relationship between internalization and disordered eating. Further research is needed to validate and better understand drive for muscularity in gay and bisexual women as it does not appear to differentially mediate the relationship between internalization and disordered eating in lesbian/bisexual women.

In the present sample, heterosexual women had higher scores than bisexual and lesbian women on the internalization subscale of the SATAQ, indicating that heterosexual women are more likely to be influenced by societal messages related to physical appearance, including body shape (Thompson et al., 2004). Despite this, lesbians did not significantly differ from heterosexual women in terms of disordered eating and the amount of variance in disordered eating accounted for by other variables in this study did not differ by sexual orientation. Thus, the data support prior research (Kozee and Tylka, 2006; Peplau et al., 2009) indicating that increased risk for self-objectification and disordered eating is not a consequence of pursuing the attraction of men (cf. Fredrickson and Roberts, 1997; Kozak et al., 2009; Wiseman and Moradi, 2010).

The largest effect size in the comparisons of heterosexual to bisexual and lesbian women was for the measure of self-esteem. More specifically, bisexual women reported the lowest self- esteem, while heterosexual and exclusively gay women did not significantly differ from each other. To our knowledge, no study has reported significantly different levels of self-esteem between bisexual, lesbian and heterosexual women. It is unclear why self-esteem was lower for the sample of bisexual women, particularly since they did not differ on most other measures and the correlation coefficients between self-esteem and other measures did not differ between the populations. It may be that these differences are driven by factors not measured in the present inventory, such as discrimination or other emotional distress. Bisexual individuals are at increased risk for a variety of negative health outcomes (Dodge et al., 2007). Bisexual women may share a more complex path from self-objectification to the development of eating disorders that has been found to be more complex for lesbians than for heterosexual women (e.g., Kozee and Tylka, 2006). It is important to note that this finding may be an artifact of an overrepresented sample of heterosexual women and a comparatively small sample of bisexual women. As bisexual women could not be parsed out in the path analysis, additional research is required to validate this finding.

### Limitations

The present study makes important contributions by validating several previous findings using a larger, more diverse sample than is typically reported. However, there were certain limitations that future research could explore and clarify. We attempted to recruit a large, diverse sample from the community and several regional universities, but the majority of respondents were under 30 years old, college students, and fairly homogenous in terms of ethnicity; this may restrict how applicable these findings are to the general population. However, these data do further clarify body image and eating disorder symptomatology exhibited by young adults. While there was a sufficiently large enough sample of women to power the analyses, heterosexual women were over-represented. For this reason, additional research on body image and eating disorder symptomatology in lesbian women is merited to validate the present findings. Bisexual men were underrepresented in the present sample, while bisexual women were disproportionally represented. While we did analyze bisexuals separately from gay men and women whenever possible, the sample sizes did not allow us to test the hypothesized model in an exclusively bisexual sub-sample. The lack of any notable differences of bisexual men may be due to their under-representation. Therefore, additional research should attempt equal representation of bisexual individuals and/or assess other dimensions of sexual orientation (e.g., by same-sex attraction, relationships, sexual behavior). Finally, due to an administration error, one item of the general internalization subscale of the SATAQ had to be replaced via imputation, which limits the validity of this scale and the ability to directly compare it to other research.

Despite these limitations, the present study contributes to emerging research demonstrating that sexual orientation is an important variable when exploring issues pertaining to body image and eating disorders. In line with previous research, this study indicates that gay men may be more prone to body and eating related disturbances than heterosexual men, but heterosexual men are not immune to body shape dissatisfaction. Drive for thinness may also play a different role in the development of disordered eating patterns in gay or bisexual men as compared to heterosexual men. The cross-sectional nature of the current study prohibits us from making any causal attributions, and the development of drive for thinness should be explored further in gay men. While lesbian and heterosexual women did not differ significantly on many items in the survey, lesbians demonstrated lower internalization of social influences of appearance as well as reduced eating disorder symptomatology; nonetheless, the model describing the path from internalization of socio-cultural standards of beauty to disordered eating was equivalent for lesbian and heterosexual women—indicating the mechanism for the development of disordered eating may be more similar for women than for men. There is now sufficient evidence to suggest that gay men are at increased risk for body dissatisfaction and eating disorder symptomatology while the same concepts have been understudied in lesbians.

## Author contributions

Chetra Yean completed this study as part of his senior thesis project. He provided initial background research, attained IRB approval, provided outreach to organizations, and was responsible for all data collection. He also contributed to the writing and editing of this manuscript. Erik M. Benau prepared, analyzed, and presented the data. He wrote up initial drafts of all sections of the manuscript. Antonios Dakanalis edited drafts of manuscript, provided statistical assistance, conducted the path analysis, and added background from his extensive knowledge on the subjects covered here. Julia M. Hormes assisted in oversight of study conceptualization and execution and was involved in the editing process of this manuscript. Julie Perone assisted in initial conceptualization and execution of the study and facilitated data collection. She also edited prior drafts of the manuscript. C. Alix Timko supervised research, advised Chetra Yean, and provided lab space for data collection. She also assisted in collecting data at two campuses from which data were collected. She coordinated and oversaw all aspects of the study and manuscript preparation.

### Conflict of interest statement

The authors declare that the research was conducted in the absence of any commercial or financial relationships that could be construed as a potential conflict of interest.

## Footnotes

^1^The community and college samples (with the three campuses combined) were not significantly different from each other on the majority of variables included in this study. The only exception was internalization of socio-cultural standards of beauty: the community sample reported significantly lower scores (*M* = 25.32, *SD* = 9.29, *Mdn* = 25.88) than the undergraduate sample [(*M* = 27.84, *SD* = 8.49, *Mdn* = 28.13), *Z* = −3.147, *U* = 39955.0, *p* = 0.002, *r* = 0.120]. Although statistically significant, it is unlikely that this is a clinically meaningful difference.

## References

[B1] Aickin M. H. (1996). Adjusting for multiple testing when reporting research results: the Bonferroni vs Holm Methods. Am. J. Public Health 86, 726 10.2105/AJPH.86.5.726 8629727 PMC1380484

[B2] Beren S. E. Hayden H. A. Wilfley D. E. Striegel-Moore R. H. (1997). Body dissatisfaction among lesbian college students. Psychol. Women Quart. 21, 431–445 10.1111/j.1471-6402.1997.tb00123.x

[B3] Bergeron D. Tylka T. L. (2007). Support for the uniqueness of body dissatisfaction from drive for muscularity among men. Body Image 4, 288–295 10.1016/j.bodyim.2007.05.002 18089275

[B4] Blashill A. J. (2011). Gender roles, eating pathology, and body dissatisfaction in men: a meta-analysis. Body Image 8, 1–11 10.1016/j.bodyim.2010.09.002 20952263

[B5] Brennan D. J. Craig S. L. Thompson D. E. (2012). Factors associated with a drive for muscularity among gay and bisexual men. Cult. Health Sex. 14, 1–15 10.1080/13691058.2011.619578 22077494

[B6] Byrne B. (2011). Structural Equation Modeling with Mplus: Basic Concepts, Application and Programming. New York, NY: Routledge

[B7] Carlat D. J. Camargo C. A. Herzog D. B. (1997). Eating disorders in males: a report on 135 patients. Am. J. Psychiatry 154, 1127–1132 9247400 10.1176/ajp.154.8.1127

[B8] Case S. (1999). Toward a butch-femme aesthetic, in Camp: Queer Aesthetics and the Performing Subject: a Reader, ed Cleto F. (Ann Arbor, MI: University of Michigan Press), 185–258

[B9] Cogan J. C. (1999). Lesbians walk the tightrope of beauty: thin is in, but femme is out. J. Lesbian Stud. 3, 77–89 10.1300/J155v03n04_11 24786429

[B10] Cohane G. H. Pope H. G. Jr. (2001). Body image in boys: a review of the literature. Int. J. Eat. Disord. 29, 373–379 10.1002/eat.1033 11285574

[B11] Cooper P. J. Taylor M. J. Cooper Z. Fairbum C. G. (1987). The development and validation of the body shape questionnaire. Int. J. Eat. Disord. 6, 485–494 10.1002/1098-108X(198707)6:4<485::AID-EAT2260060405>3.0.CO;2-O

[B12] Cooper Z. Fairburn C. G. (2011). The evolution of “enhanced” cognitive behavior therapy for eating disorders: Learning from treatment nonresponse. Cogn. Behav. Practice 18, 394–402 10.1016/j.cbpra.2010.07.007 23814455 PMC3695554

[B13] Dakanalis A. Di Mattei V. E. Pagani Bagliacca E. Prunas A. Sarno L. Riva G. (2012). Disordered eating behaviors among italian men: objectifying media and sexual orientation differences. Eat Disord. 20, 356–367 10.1080/10640266.2012.715514 22985233

[B14] Dakanalis A. Timko A. C. Madeddu F. Volpato C. Clerici M. Riva G. (2013a). Are the male body dissatisfaction and drive for muscularity scales reliable and valid instruments. J. Health Psychol. [Epub ahead of print]. 10.1177/1359105313498108 23988678

[B15] Dakanalis A. Zanetti M. Riva G. Clerici M. (2013b). Psychosocial moderatorsof the relationship between body dissatisfaction and symptoms of eatingdisorders: a look at a sample of young Italian women. Eur. Rev. App. Psychol. 63, 323–334 10.1016/j.erap.2013.08.001

[B16] Dakanalis A. Timko C. A. Clerici M. Zanetti M. A. Riva G. (2014). Comprehensive examination of the trans-diagnostic cognitive behavioral model of eating disorders in males. Eat. Behav. 15, 63–67 10.1016/j.eatbeh.2013.10.003 24411752

[B17] Dakanalis A. Riva G. (2013). Current considerations for eating and body-related disorders among men, in Handbook on Body Image: Gender Differences, Sociocultural Influences and Health Implications, eds Sams L. B. Keels J. A. (New York, NY: Nova Science Publishers), 195–216

[B18] Dodge B. Sandfort T. G. Firestein B. (2007). A review of mental health research on bisexual individuals when compared to homosexual and heterosexual individuals, in Becoming Visible: Counseling Bisexuals Across the Lifespan. ed Firestein B. (New York, NY: Columbia University Press), 28–51

[B19] Drucker D. J. (2012). Marking sexuality from 0–6: the kinsey scale in online culture. Sex. Cult. 16, 241–262 10.1007/s12119-011-9122-1

[B20] Duggan S. J. McCreary D. R. (2004). Body image, eating disorders, and the drive for muscularity in gay and heterosexual men: the influence of media images. J. Homosex. 47, 45–58 10.1300/J082v47n03_03 15451703

[B21] Feldman M. B. Meyer I. H. (2007). Eating disorders in diverse lesbian, gay, and bisexual populations. Int. J. Eat. Disord. 40, 218–226 10.1002/eat.20360 17262818 PMC2080655

[B22] Fitzsimmons-Craft E. E. (2011). Social psychological theories of disordered eating in college women: review and integration. Clin. Psychol. Rev. 31, 1224–1237 10.1016/j.cpr.2011.07.011 21903047

[B23] Fredrickson B. L. Roberts T. A. (1997). Objectification theory. Psychol. Women Quart. 21, 173–206 10.1111/j.1471-6402.1997.tb00108.x

[B24] Garner D. M. (1991). Eating Disorder Inventory-2: Professional Manual. Odessa, FL: Psychological Assessment Resources

[B25] Garner D. M. Garfinkel P. E. (1979). The eating attitudes test: an index of the symptoms of anorexia nervosa. Psychol. Med. 9, 273–279 10.1017/S0033291700030762 472072

[B26] Grogan S. (2008). Body image: Understanding Body Dissatisfaction in Men, Women, and Children, 2 Edn. New York, NY: Routledge

[B27] Grogan S. Richards H. (2002). Body image focus groups with boys and men. Men Masc. 4, 219–232 10.1177/1097184X02004003001

[B28] Herzog A. (2011). U.S. Adults Overestimate Homosexual Population by As Much As Tenfold [Online]. cnsnews.com. Available online at: http://cnsnews.com/news/article/us-adults-overestimate-homosexual-population-much-tenfold (Accessed on March 22, 2013).

[B29] Holm S. (1979). A simple sequentially rejective multiple test procedure. Scand. J. Statistics 6, 65–70

[B30] Hunt C. J. Gonsolkorale K. Nosek B. A. (2012). LInks between psychosocial variables and body dissatisfaction in homosexual men: differential relations with the drive for muscularity and the drive for thinness. Int. J. Mens Health. 11, 127–136 10.3149/jmh.1102.127

[B31] Jankowski G. S. Diedrichs P. C. Halliwell E. (2013). Can appearance conversations explain differences between gay and heterosexual men's body dissatisfaction. Psychol. Men Masc. 10.1037/a0031796

[B32] Jones R. Malson H. (2013). A critical exploration of lesbian perspectives on eating disorders. Psychol. Sex. 4, 62–74 10.1080/19419899.2011.603349

[B33] Kinsey A. C. Pomeroy W. B. Martin C. E. (1948). Sexual Behavior in the Human Male. Philadelphia, PA: W.B. Saunders and Co.

[B34] Koff E. Lucas M. Migliorini R. Grossmith S. (2010). Women and body dissatisfaction: does sexual orientation make a difference. Body Image 7, 255–258 10.1016/j.bodyim.2010.03.001 20395185

[B35] Kozak M. Frankenhauser H. Roberts T.-A. (2009). Objects of desire: objectification as a function of male sexual orientation. Psychol. Men Masc. 10, 225–230 10.1037/a0016257

[B36] Kozee H. B. Tylka T. L. (2006). A test of objectification theory with lesbian women. Psychol. Women Quart. 30, 348–357 10.1111/j.1471-6402.2006.00310.x

[B37] Levine M. P. Murnen S. K. (2009). “Everybody knows that mass media are/are not [pick one] a cause of eating disorders:” a critical review of evidence for a causal link between media, negative body image, and disordered eating in females. J. Soc. Clin. Psychol. 28, 9–42 10.1521/jscp.2009.28.1.9

[B38] Levitt H. Hiestand K. (2004). A quest for authenticity: contemporary butch gender. Sex Roles 50, 605–621 10.1023/B:SERS.0000027565.59109.80

[B39] Lewis I. Watson B. White K. M. (2009). Internet versus paper-and-pencil survey methods in psychological experiments: equivalence testing of participant responses to health-related messages. Aust. J. Psychol. 61, 107–116 10.1080/00049530802105865

[B40] Li N. P. Smith A. R. Griskevicius V. Cason M. J. Bryan A. (2010). Intrasexual competition and eating restriction in heterosexual and homosexual individuals. Evol. Hum. Behav. 31, 365–372 10.1016/j.evolhumbehav.2010.05.004 20835352 PMC2935594

[B41] Mackinnon D. (2011). Integrating mediators and moderators in research design. Res. Soc. Work Pract. 21, 675–681 10.1177/1049731511414148 22675239 PMC3366634

[B42] McCreary D. R. Sasse D. K. (2000). An exploration of the drive for muscularity in adolescent boys and girls. J. Am. Coll. Health 48, 297–304 10.1080/07448480009596271 10863873

[B43] Morrison M. A. Morrison T. G. Sager C.-L. (2004). Does body satisfaction differ between gay men and lesbian women and heterosexual men and women?: a meta-analytic review. Body Image 1, 127–138 10.1016/j.bodyim.2004.01.002 18089146

[B44] Muthén L. Muthén B. (2010). MPlus User's Guide, 6 Edn. Los Angeles, CA: Muthén & Muthén

[B45] Olivardia R. Pope H. G. Jr. Borowiecki J. J. Iii Cohane G. H. (2004). Biceps and body image: the relationship between muscularity and self-esteem, depression, and eating disorder symptoms. Psychol. Men Masc. 5, 112–120 10.1037/1524-9220.5.2.112

[B46] Peplau L. A. Frederick D. A. Yee C. Maisel N. Lever J. Ghavami N. (2009). Body image satisfaction in heterosexual, gay, and lesbian adults. Arch. Sex. Behav. 38, 713–725 10.1007/s10508-008-9378-1 18712469

[B47] Rosenberg M. (1965). Society and the Adolescent Self-Image. Princeton, NJ: Princeton University Press

[B48] Russell C. J. Keel P. K. (2002). Homosexuality as a specific risk factor for eating disorders in men. Int. J. Eat. Disord. 31, 300–306 10.1002/eat.10036 11920991

[B49] Sell R. L. (1997). Defining and measuring sexual orientation: a review. Arch. Sex. Behav. 26, 643–658 10.1023/A:1024528427013 9415799

[B50] Soh N. L. Touyz S. W. Surgenor L. J. (2006). Eating and body image disturbances across cultures: a review. Eur. Eat. Disord. Rev. 14, 54–65 10.1002/erv.67819703916

[B51] Stice E. (1994). Review of the evidence for a sociocultural model of bulimia nervosa and an exploration of the mechanisms of action. Clin. Psychol. Rev. 14, 633–661 10.1016/0272-7358(94)90002-7

[B52] Stice E. Shaw H. E. (2002). Role of body dissatisfaction in the onset and maintenance of eating pathology: a synthesis of research findings. J. Psychosom. Res. 53, 985–993 10.1016/S0022-3999(02)00488-9 12445588

[B53] Stice E. Shaw H. Marti C. N. (2007). A meta-analytic review of eating disorder prevention programs: encouraging findings. Annu. Rev. Clin. Psychol. 3, 207–231 10.1146/annurev.clinpsy.3.022806.091447 17716054

[B54] Striegel-Moore R. H. Rosselli F. Perrin N. Debar L. Wilson G. T. May A. (2009). Gender difference in the prevalence of eating disorder symptoms. Int. J. Eat. Disord. 42, 471–474 10.1002/eat.20625 19107833 PMC2696560

[B55] Thompson J. K. Van Den Berg P. Roehrig M. Guarda A. S. Heinberg L. J. (2004). The sociocultural attitudes towards appearance scale-3 (SATAQ-3): development and validation. Int. J. Eat. Disord. 35, 293–304 10.1002/eat.10257 15048945

[B56] Wiseman M. C. Moradi B. (2010). Body image and eating disorder symptoms in sexual minority men: a test and extension of objectification theory. J. Couns. Psychol. 57, 154–166 10.1037/a0018937 21133567

[B57] Yelland C. Tiggemann M. (2003). Muscularity and the gay ideal: body dissatisfaction and disordered eating in homosexual men. Eat. Behav. 4, 107–116 10.1016/S1471-0153(03)00014-X 15000974

